# The Potential of Mesenchymal Stem Cells to Treat Systemic Inflammation in Horses

**DOI:** 10.3389/fvets.2019.00507

**Published:** 2020-01-21

**Authors:** Elizabeth S. MacDonald, Jennifer G. Barrett

**Affiliations:** Marion duPont Scott Equine Medical Center, Virginia Maryland College of Veterinary Medicine, Virginia Tech, Leesburg, VA, United States

**Keywords:** equine, inflammation, endotoxemia, bone marrow derived mesenchymal stem cells, immunomodulation

## Abstract

One hallmark of mesenchymal stem cells (MSCs) is the ability to differentiate into multiple tissue types which assists in tissue regeneration. Another hallmark of MSCs is their potent anti-inflammatory and immunomodulatory properties and the potential to treat inflammatory, immune-mediated, and ischemic conditions. In equine practice, MSCs have shown efficacy in the treatment of musculoskeletal disorders such as tendinopathy, meniscal tears and cartilage injury. However, there are many equine disease processes and conditions that may benefit from the immunomodulatory properties of MSCs. Examples include conditions associated with overwhelming acute inflammatory response such as systemic inflammatory response syndrome to chronic diseases characterized by a prolonged low level of inflammation such as equine asthma and recurrent uveitis. For the acute inflammatory response processes, there is often high morbidity and mortality with no effective immunomodulatory treatment to prevent the overwhelming synthesis of proinflammatory mediators. For chronic inflammatory disease processes, frequently long-term corticosteroid treatment is the therapeutic mainstay, with serious potential complications. Thus, there is an unmet need for alternative anti-inflammatory treatments for both acute and chronic illnesses in horses. While MSCs show promise for such conditions, much research is needed before a clinically safe and effective treatment will be available. Optimal MSC tissue source, patient vs. donor source (autologous vs. allogeneic) and cell growth conditions need to be determined for each problem. For immediate use, allogeneic MSC treatments is preferable, but immune tolerance and adequate safety require further study. MSC collection and cryopreservation from horses before they are injured or ill, whether from umbilical cord tissue, bone marrow or adipose might become more widespread. Once these fundamental approaches to treating specific diseases with MSCs are determined, the route of administration, dose and timing of administration also need to be studied. To provide a framework for development of MSC immunomodulatory treatments, this article reviews the current understanding of equine MSC anti-inflammatory and immunomodulatory properties and proposes how MSC therapy may be further developed to treat acute onset systemic inflammatory processes and chronic inflammatory diseases.

## Defining Stem Cells

Stem cells are unique in their ability to differentiate into multiple tissue lineages and their capacity for generating numerous cells through multiple cell divisions. Stem cells are classified as either embryonic or adult in origin. Embryonic stem cells are derived from the blastocyst inner cell mass and are pluripotent—capable of differentiating into almost all the cells of the body, regardless of germ layer of origin. Adult stem cells can be isolated from almost every tissue of the body, are termed multipotent due to having more limited differentiation potential and are thought to function in the maintenance and repair of the tissue of origin, as well as other tissues in the body. Mesenchymal stem cells are adult stem cells that are multipotent cells of non-hematopoietic origin that have the ability to differentiate into adipocytes, chondrocytes, and osteocytes, also known as tri-lineage differentiation. MSCs reside in many tissues, including bone marrow, adipose tissue, brain, lung, and liver (1). The most common tissue sources for equine mesenchymal stem cells are bone marrow, adipose, and umbilical cord blood (2–5).

The Mesenchymal and Tissue Stem Cell committee has proposed a set of standards to define human MSCs. MSCs must be plastic adherent when maintained in standard culture conditions. Greater than 95% of the MSC population must express cluster of differentiation (CD) 105, CD73, and CD90. The cells must lack expression of CD45, CD34, CD14 or CD11b, CD79α or CD19, and human leukocyte antigen (HLA) class II. Finally, the cells must be able to differentiate into osteoblasts, adipocytes, and chondroblasts under standard *in vitro* differentiating conditions (6).

A set of standards has not been defined for the equine MSC thus far. Equine MSCs derived from bone marrow are adherent to plastic, exhibit the ability to differentiate into osteoblasts, adipocytes, and chondrocytes and are CD90 positive (7). More importantly, they exhibit expression of CD105, CD44, and CD90 with low or negative expression of CD34 and major histocompatibility complex II (MHC-II) (5). Differences have been noted with another study showing equine bone marrow derived MSCs are heterogenous in MHC-II expression. Variation in expression of MHC-II is seen through multiple passages, as well (8). One study of adipose-derived MSCs produced mixed results, showing an increased expression of CD44 with increased number of passages in a small number of samples (9). These differences demonstrate that despite similarities to the human definition of stem cells, making uniform conclusions about the true definition of an equine mesenchymal stem cell is difficult. Based on the research performed to this point, De Schauwer et al. proposed the definition of an equine MSC as (1) plastic adherent, (2) multipotent and capable of trilineage differentiation, and (3) positive expression for CD29, CD44, and CD90 expression and negative for CD14, CD79α, and MHC-II (10).

The mechanism of action through which stem cells exhibit their biologic effects has not been fully characterized. In using MSCs for tissue regeneration, it was thought that the MSCs may either differentiate directly into the affected tissue cells or bioactive molecules released from the damaged cell stimulate the MSCs which enhance the activity of the resident cells for repair (11). MSCs have a large number of interactions with the surrounding cells that include cell-to-cell contact, mediator secretion, and the production of extracellular vesicles (12). MSCs are also known to be able to secrete factors that enhance angiogenesis, recruit local stem cells, and they interact with both the innate and adaptive immune system (13–15). Previous work has demonstrated that intravenously administered MSCs rapidly accumulate in the lungs and are short-lived (16). The seemingly short survival of MSCs does not appear to interfere with their biologic effects as these effects are seen for much longer than 24 h. In a murine model, human umbilical cord MSCs injected intravenously are cleared from the lungs within 24 h. Phagocytosis of MSCs by monocytes and neutrophils contribute to clearance. Phagocytosis of MSCs appears to induce functional and phenotypic changes in monocytes which modulates their cellular response (17).

In the equine patient, the research focus has been on the use of MSCs for tissue regeneration and healing. This is partly based on MSCs ability to differentiate *in vitro* to the desired tissue type, but this may not reflect what occurs *in vivo*. Labeling studies in the horse have shown that the majority of MSCs directly injected into a tendon or a joint to treat cartilage injury are lost from the injection site over time (18). MSCs may either differentiate into the required cell type over time or exhibit paracrine effects to stimulate healing prior to leaving the area, or both. In an equine study, intrathecal injection of allogeneic adipose-derived MSCs in three horses with cervical vertebral compressive myelopathy did not result in detectable MSCs at 7 or 15 days at the site of injury (19). The failure to engraft at the site of injection may be related to the allogeneic or tissue source of cells, how the cells were prepared for injection, in addition to how MSCs might normally function in the body. If MSCs are phagocytosed by monocytes (17), as demonstrated in murine models, then the positive effects may be from the MSCs inducing the distribution of monocytes with immunoregulatory properties through the body. Much research is needed to better understand the signals for MSC recruitment, migration and retention, also known as stem cell “homing.” There are documented reports in other species which MSCs appear to differentiate into the required tissue for healing. In models of spinal cord injury, implanted MSCs differentiate into various neural cell types to participate in cord regeneration (20). In cardiac muscle following infarction, MSCs differentiate into endothelial cells, undergo cardiomyogenic differentiation, and fuse with existing muscle cells to help prolong the survival of the intrinsic cells (21). In addition to the strong evidence of MSCs ability to differentiate and aid in tissue regeneration, MSCs are known also to have anti-inflammatory and immune-enhancing response (22).

## Mesenchymal Stem Cells Modulate Inflammation

**Figure 1:**
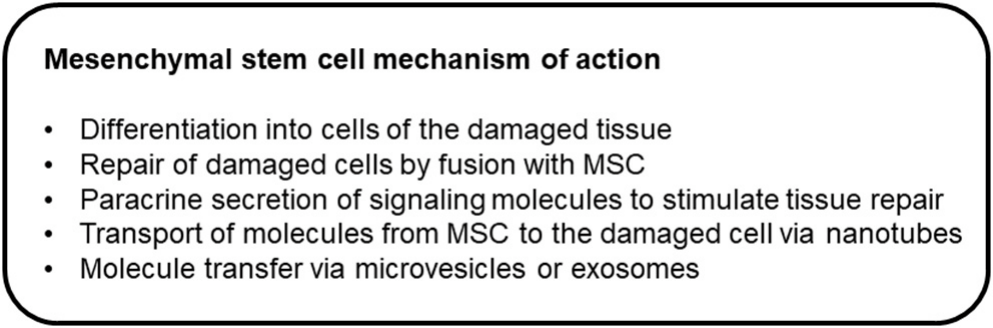
Mechanisms of action through which mesenchymal stem cells may assist healing or treat disease.

Ideally, MSCs would express low concentrations of MHC-I and not express MHC-II (1), which would contribute to immune tolerance. Equine MSCs have been shown to express MHC-I, and they are heterogeneous for MHC-II expression (8, 23, 24). MSCs produce many cytokines, growth factors, chemokines, and immunomodulatory proteins (25). Through these mechanisms, they can induce angiogenesis, stimulate intrinsic cells to regenerate, and induce apoptosis (26). MSCs induce apoptosis of activated T cells, decrease T cell proliferation, and alter T cell phenotype (22). MSCs also alter lymphocyte proliferation by inducing the expansion of regulatory T cells (27). Direct cell contact with MSCs is not required for these effects (28), which suggest secreted soluble factors or extracellular vesicles (exosomes) are exerting these effects. Figure 1 provides a proposed list of mechanisms by which MSCs may assist healing or treat disease.

In humans and rodents, MSCs act through secretion of soluble factors or direct cell-to-cell contact to affect T cells, Natural Killer (NK) cells, B cells, and dendritic cells (15) *in vitro*. MSCs induce apoptosis of activated T cells, induce cell cycle arrest, decrease T cell proliferation, and alter T cell phenotype. They target all T cell subsets (CD4+, CD8+) equally (29). Equine bone marrow derived MSCs exhibit a dose dependent immunosuppression of T cell subsets (30). Both autologous and allogeneic MSCs exhibit equivalent degrees of suppression of T cell proliferation (31). Suppression of T cell function by MSCs may utilize many different pathways, but the cyclooxygenase pathway appears to play an important role (31).

MSCs alter NK cell phenotype and suppress cytokine-induced proliferation of NK cells (15, 32). They have the ability to promote the survival and inhibit the proliferation and maturation of B cells by arresting them in the G_0_/G_1_ phase of the cell cycle (33). MSCs can also modulate dendritic cell maturation, differentiation, and function (34). In mice, it has been suggested that MSCs decrease the proliferation of B cells (26). Currently, the interaction of equine MSCs with B cells and dendritic cells has not been described (22).

Stem cells need to be activated in some manner as they do not release immunomodulatory factors after standard *in vitro* culture (26). Quiescent MSCs in G_0_ of the cell cycle derived from multiple sources do not alter lymphocyte proliferation or secrete mediators except for transforming growth factor–β (TGF-β) (35). Exposure to pro-inflammatory molecules such as interferon gamma (IFN-γ), tumor necrosis factor alpha (TNF-α), IL-1, and lipopolysaccharide (LPS) helps target MSCs to the site of injury (“homing”) and activate them to start secreting their bioactive markers (25). MSC homing to the site of injury is aided by VCAM-1 and E selectin activated by injured endothelial cells (36). The exact mechanism of MSC modulation is not known, but when human and rodent MSCs are activated they express a number of inhibitory factors including nitric oxide (NO), indoleamine-pyrrole 2,3-dioxygenase (IDO), interleukin-10 (IL-10), TGF-β, TNFα-stimulated gene-6 (TSG-6), and prostaglandin E_2_ (PGE_2_). They also express surface molecules intracellular adhesion molecule 1 (ICAM-1) and vascular cell adhesion molecule (VCAM) and a number of growth factors including endodermal growth factor (EGF), fibroblast growth factor (FGF), platelet derived growth factor (PDGF), vascular endothelial growth factor (VEGF), and stromal cell derived factor-1 (SDF-1) (25, 36, 37). Phenotype, differentiation potential, and gene expression are altered by *in vitro* passage which may influence the response that MSCs have in their environment (38). Equine MSCs decrease production of TNF-α and IFN-γ while showing increased production of PGE_2_ and IL-6 (35). MSCs with MHC-II expression treated with TNF-α showed no increase in MHC-II expression. However, they did exhibit increased expression of MHC-I, IL-6, and IL-8 (24). MHC-II expression was downregulated by exposure to IL-1β (24). These findings further support the fact that MSCs must be exposed to inflammatory mediators to exhibit their immunosuppressive abilities.

With the evidence that MSCs require exposure to inflammatory mediators to activate their immunosuppressive properties, the environment that the MSCs are injected into may play a role in their immunomodulatory response. After priming human MSCs with IFN-γ and TNF-α, the MSCs were more effective at inhibiting T cell proliferation when compared to unprimed MSCs (39). Priming of equine MSCs with cytokines can induce significant upregulation of VCAM-1, IDO, inducible nitric oxide synthase (iNOS), IL-6, and down regulation of IL-10 *in vitro* (40). Bone marrow derived equine MSCs exposed to IFN-γ and TNF-α showed increased expression of VCAM-1, cyclooxygenase-2 (Cox-2), IDO, iNOS, and IL-6 when compared to MSCs exposed to inflammatory synovial fluid (41). Gene expression of MHC-II was also upregulated by exposure to IFN-γ and TNF-α (41). While not significant, there was a general trend that MSCs that were MHC-II positive had a greater magnitude of response to toll-like receptor 4 (TLR4) stimulation and an increased ability to suppress T cell proliferation (42). *In vitro*, priming equine MSCs with IFN-γ showed better ability to suppress T-cell proliferation when compared to naïve MSCs. This effect was also maintained following exposure to inflammatory macrophages (43). Priming MSCs prior to injection may ensure that the best response is achieved as some treatment environments may not adequately activate the cells. However, the agent used for priming may affect MSC response. All of these studies on priming MSCs have occurred *in vitro* which does not always reflect what occurs *in vivo*.

Exposing equine bone marrow derived MSCs to TGF-β2 has been shown to downregulate MHC-I and MHC-II surface expression when compared to controls. It also partially blocked the IFN-γ induced upregulation of MHC (44). Exposing donor MSCs to TGF-β2 prior to allogeneic use may decrease the potential for immune reaction, however individual variation was noted. The effect of TGF-β2 was dependent on the baseline expression of MHC and donor animals (44). Further *in vitro* evidence suggests that cytokine priming has a negative impact on the MSCs viability and differentiation when compared to MSCs primed in an inflammatory synovial fluid (45).

**Figure 2:**
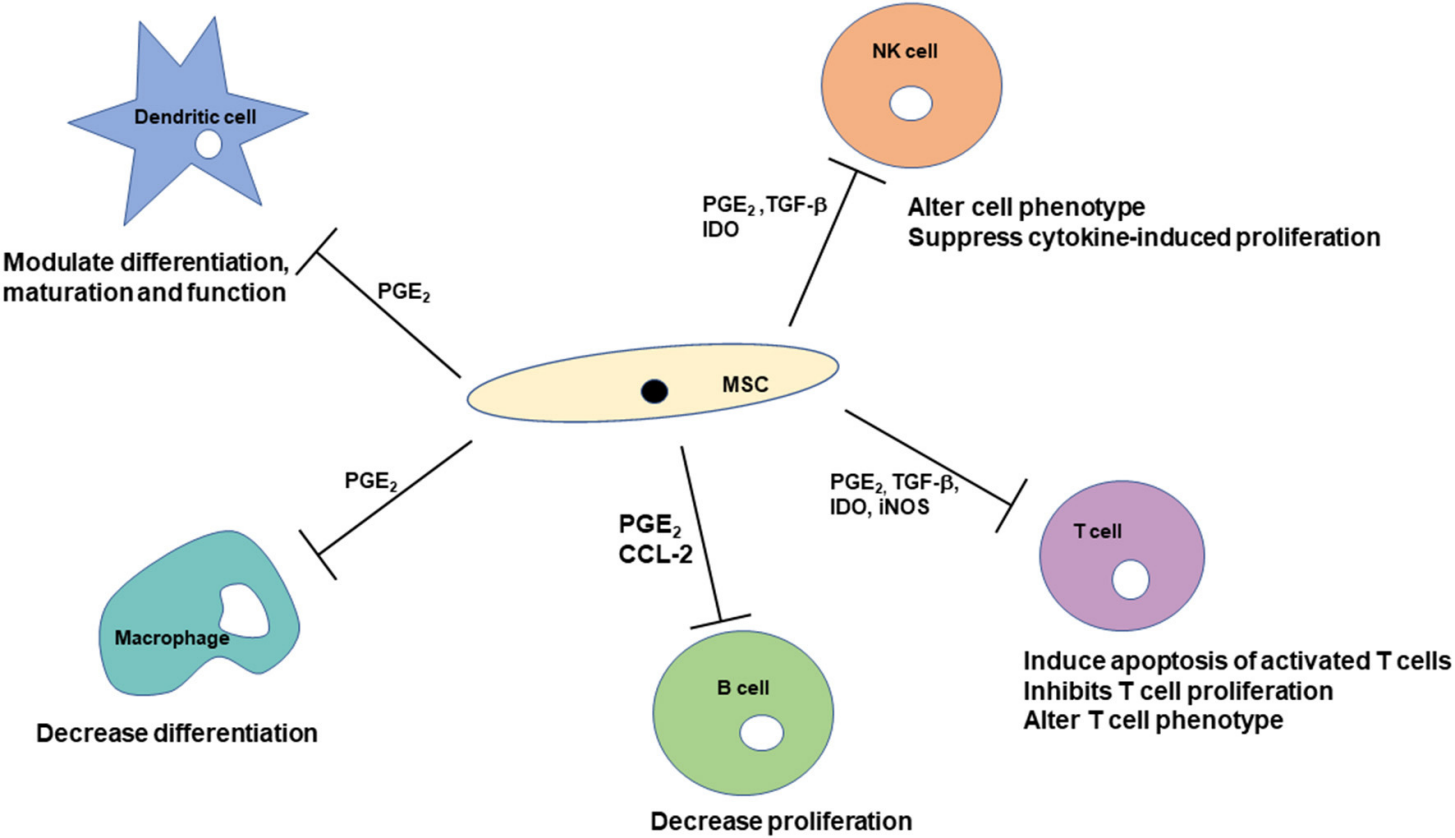
Mechanisms through which mesenchymal stem cells can affect cells of the immune system. Immunomodulatory effects are modulated by soluble factors as well as cell-to-cell contact. The immunomodulatory effects include suppression of cell proliferation, modulating differentiation and maturation, and alteration of cell phenotypes.

The sources of MSCs produce slightly different responses. MSCs derived from bone marrow and umbilical cord blood produce nitric oxide, and MSCs derived from adipose tissue do not (35). The pathways through which MSCs inhibit T cell proliferation are also different between tissue sources. MSCs from bone marrow and umbilical cord blood inhibit T cell proliferation through cell cycle arrest while MSCs from adipose tissue and umbilical cord tissue inhibit T cell proliferation via induction of apoptosis (46). Variation in MHC-II expression of MSCs isolated from bone marrow aspirates from the same horse at different time points has been reported (8). Figure 2 provides an overview and compilation of the impact of MSCs on cells of the immune system.

## Clinical Applications for MSCs to Treat Medical Conditions in the Horse

Based on our current understanding of the biology of MSCs, there is substantial potential to harness the immunomodulatory properties of stem cells to treat medical conditions in the horse. In human medicine, the targets for development of MSC treatments have been inflammatory, immune-mediated, and ischemic diseases (11). Numerous human clinical trials are ongoing in the treatment of cardiac, respiratory, gastrointestinal, ocular, and neurologic disorders as well as neoplasia. All of the data generated from the thousands of human studies can help determine which therapeutic approaches to which conditions might be best studied for the horse. Areas of unmet need, and potential therapeutic use of MSCs for equine patients include endotoxemia, inflammatory bowel disease, equine asthma, and recurrent uveitis. The following sections will review the animal model and human literature with respect to each type of condition, and describe current research efforts in equine medicine.

## Endotoxemia in the Horse and MSCs

Endotoxemia affects 20–30% of human patients in intensive care units and accounts for more than 200,000 human deaths per year in the United States (47). In humans, endotoxemia has been correlated with the development of multiple organ failure and death (48). In horses, colic remains the leading cause of death in the adult, and reports show that mortality is closely related to the degree of endotoxemia (49, 50). Adult horses that present to referral hospitals for colic, 30–40% have detectable endotoxin in their circulation (51). Higher concentrations of endotoxin at admission and intra-operatively have been associated with increased mortality (52). Endotoxemia can be caused by any gram-negative infection. The gastrointestinal tract is the most common source of endotoxin, and strangulating or obstructive lesions are the most common cause. Endotoxemia is a common sequela of colitis, peritonitis, pleuropneumonia, and metritis. Up to 50% of foals that present for septicemia have detectable circulating endotoxin (53).

Clinical signs of endotoxemia in the horse are non-specific and dose-dependent. Leukopenia is usually observed early in the disease process (54). Initial clinical signs of tachypnea, fever, depression, and inappetence are mediated by the initial cytokine release (55). Clinical signs progress as a release of iNOS causes local release of NO, production of thromboxane A_2_ and prostaglandin F_2_α leading to the development of hypotension. Four to six hours after exposure, a second wave of tachypnea and tachycardia occurs that is associated with fever. Production of prostaglandin I_2_ and PGE_2_ contribute to the development of hypotension (55). In this time period, the activation of ICAM-1 and VCAM-1 along with β2 integrins and other chemokines activates neutrophil binding to the endothelial wall. The neutrophils then follow the chemotactic gradient and extravasate into the affected tissue. Arachidonic acid (AA) is also metabolized to leukotrienes that promote fluid leakage across capillaries, and act as potent chemoattractants for neutrophils and potent bronchoconstrictors and vasoconstrictors (56). These systemic changes can lead to a number of other complications including thrombophlebitis and laminitis (57).

To date, there is no single effective treatment for equine endotoxemia. The current goals of therapy are to reduce or prevent the movement of endotoxin into circulation, neutralize circulating endotoxin, prevent or reduce interaction with inflammatory cells, prevent the synthesis of proinflammatory mediators, and supportive care.

A number of immunosuppressive properties of stem cells have been recognized to date. They strongly inhibit the proliferation of T cells and also B cells while inducing expansion of the regulatory T cells (22). Stem cells can inhibit the differentiation of monocytes to immature dendritic cells and prevent the production of TNF-α by dendritic cells. Stem cells also inhibit the proliferation of resting NK cells and therefore reduce cytokine release (22). Multiple studies support the positive role that stem cells play in reducing the effects of endotoxin in mice. Endotoxin injection in mice causes a systemic inflammatory response and alterations in lung structure and function. Untreated mice that received endotoxin exhibited vascular congestion and an increase in neutrophils within the lungs. Mice that received 5 × 10^5^ bone marrow derived MSCs intravenously after endotoxin demonstrated a decreased response in circulating proinflammatory cytokines (58).

Cecal ligation and puncture (CLP) in rodents is a useful model of endotoxemia and sepsis with a high morbidity and mortality rate. After CLP, the initial inflammatory response is characterized by the release of pro-inflammatory cytokines (59). Treatment with 2.5 × 10^5^ MSCs intravenously 6 h after CLP led to decreased production of plasma IL-6, IL-1β, and IL-10 (60). Intraperitoneal administration of human adipose derived MSCs (10^5^-10^6^) decreased inflammatory mediators (TNF-α, IL-6, IL-1β, and IFN-γ) when compared to controls (61). In addition, administration of MSCs following CLP has demonstrated a reduction in systemic levels of creatinine, reduced the number of apoptotic cells in the kidney (60) as well as a reduction in the number of apoptotic and necrotic cells in the spleen (62). The kidney is often one of the first affected organs during sepsis. These findings suggest that the stem cells may provide a protective role to the organs.

MSCs appear to modulate the host's abilities to clear bacterial infection and decrease the mortality rate. Significantly fewer colony forming units were present in the spleen of septic mice treated intravenously with MSCs compared to controls after undergoing CLP (60). Mice treated with MSCs following CLP exhibited a 24% mortality rate at 28 h after the procedure while the controls receiving sham treatment exhibited a 45% mortality rate (60). Treatment with MSCs alone appears to improve mortality when compared to controls. Septic mice treated with antimicrobial therapy in addition to treatment with MSCs exhibited significant improvement in survival when compared to the sham treated mice (60). *In vitro*, equine MSCs have inhibited the growth of *Escherichia coli* and *Staphylococcus aureus* when cultured in direct and indirect contact (63).

Further evidence of antimicrobial properties of MSCs was seen in mice with experimentally induced chronic *S. aureus* infections treated with intravenous adipose derived stem cells (1 × 10^6^ cells/mouse). A combination of antimicrobial therapy and administration of TLR3 ligand-activated MSCs significantly reduced bacterial burden at the wound site. Non-activated MSCs alone did not significantly reduce bacterial burden (64). *In vitro* studies have demonstrated that non-activated adipose MSCs did have a direct killing effect on *S. aureus* (64). This demonstrates that the MSCs may behave differently *in vivo* compared to *in vitro*. Repeated intravenous injections of allogeneic activated MSCs [2 × 10^6^ cells/kilogram (kg) of body weight] also had positive effects in canine wounds with multi-drug resistant bacteria (64). The patients remained on antimicrobial therapy during stem cell treatment. Antimicrobials may have a synergistic effect with activated MSCs.

The potential benefit of MSC therapy for sepsis was documented in a meta-analysis showing that overall odds of death in rodents with sepsis was reduced by treatment with MSCs. This effect was reported to be maintained over a range of time periods as well (65). Most of the studies included in the meta-analysis (65) did not address late administration of MSCs (after 6 h of illness) and there was a wide variation in doses, timing of administration, and severity of disease.

Assessment of human MSCs in an *in vitro* endotoxemia model demonstrated similar findings that MSCs were able to inhibit lymphocyte proliferation in LPS stimulated cells (66). A clinical trial for the use of allogeneic stem cells to treat septic shock in humans has finished phase I trial. Intravenous administration of 250 million MSCs was safe and tolerated in patients with septic shock with no adverse reactions (67). Higher doses (4 × 10^6^ cells/kg) had an effect on a variety of pro- and anti-inflammatory mediators in healthy patients with experimentally induced endotoxemia (68). Overall there is strong evidence that MSCs may be beneficial in the treatment of endotoxemia.

## Respiratory System and MSCs

A number of acute and chronic respiratory diseases affect humans and equines. Horses are not prone to high morbidity and mortality from chronic respiratory diseases, but respiratory disease can affect athletic performance. The lung is very sensitive to endotoxin, and acute lung injury is often caused by sepsis in very ill patients (69).

Acute respiratory distress syndrome (ARDS) or acute lung injury (ALI) is caused by an acute uncontrolled inflammatory process that disrupts the lung endothelial and epithelial barrier. ARDS/ALI is characterized by the loss of alveolar-capillary membrane integrity, excessive neutrophil migration, and release of pro-inflammatory mediators (69) that results in pulmonary edema and respiratory failure if not controlled. This can be caused by primary lung injury or can be secondary to other problems such as trauma, sepsis, or endotoxemia. Despite supportive care, it can carry up to a 40% mortality rate in humans (70). In foals, there is a 60–70% survival rate from ALI (71), and it is often secondary to systemic inflammatory response syndrome or pulmonary infection. Following the initiating event, there is an uncontrolled release of inflammatory mediators. Activated neutrophils and alveolar macrophages move into the pulmonary tissue where they release oxidants, reactive oxygen species, and more cytokines to perpetuate the damage. The alveolar endothelial and epithelial barrier breaks down, leading to pulmonary edema and the initial onset of respiratory distress. Within 24 h, there is a fibroproliferative response characterized by necrosis of type I pneumocyte and proliferation of type II pneumocytes to restore the epithelial barrier. The overall effect is severe hypoxemia with hypo- or hypercapnia due to ventilation-perfusion mismatch (72). Treatment is limited to supportive care and anti-inflammatory drugs. The mainstay of treatment in humans is ventilation (70), but this can be a challenge in equine patients.

Intrapulmonary administration of bone marrow-derived MSCs showed a promising decrease in the severity of injury to the lung in a murine model of endotoxin-induced ALI (73). At 24 h after administration, decreased lung edema was observed. At 48 h, further decreased lung edema, decreased protein infiltration into the lung, and a reduction in the presence of TNF-α in the bronchoalveolar lavage fluid were observed. At 72 h, survival in the MSCs group was 64% compared to 18% in the control (saline) group without any additional supportive treatment (73). Intravenous injection of stem cells demonstrated similar results with a decrease in the neutrophil accumulation within the lung and a reduction in the presence of TNF-α (74). Intrabronchial administration of varying doses of human stem cells (4, 10, or 40 million) also demonstrated similar results in a sheep model of endotoxin-induced ARDS. The treated group showed significant improvement in lung function 2 h after treatment when compared to the control group that never returned to baseline values in the 6-h monitoring period. The positive improvements were also confirmed by histopathology (75). Intravenous administration of human menstrual blood-derived SCs showed an increase in IL-10 at 48 h post-treatment while IL-1β decreased, suggesting attenuation of inflammation (76). These models provide evidence that the MSCs shift the response after injury from a proinflammatory response to an anti-inflammatory response. ALI and ARDS are not documented as frequently in adult horse, this may be a benefit to the equine neonatal population. Further research is needed, but stem cells show promise in the therapy of ALI and ARDS. While ALI and ARDS are acute problems, there are chronic conditions such as equine asthma, that may benefit from stem cell therapy.

Equine asthma syndrome is a term that encompasses a spectrum of inflammatory diseases of the airways. This includes recurrent airway obstruction (RAO), a more chronic and severe form of respiratory disease and inflammatory airway disease (IAD) a mild inflammatory response with limited pulmonary dysfunction (77). Equine asthma is an inflammatory disease process characterized by excessive mucus production, neutrophil accumulation, bronchial hyperreactivity, and reversible bronchospasm. Hypersensitivity to inhaled molds and other organic dusts is believed to be the initiating cause with a prevalence from 2 to 80% depending on the inclusion criteria of the study (78). The underlying immunologic mechanisms that lead to pulmonary inflammation have not been fully described. In affected horses, any possible stimulus (molds or dust) activates the lymphocytes, leading to neutrophil recruitment and inflammation within the airway. There is an influx of neutrophils into the airways with an increase in CD4+ T cells in bronchial alveolar lavage fluid (79). Different studies show inconsistent results whether a T helper 1 or 2 response predominates, but the cytokines involved in the pathogenesis include IL-4, IL-5, IL-8, IL-13, and IFN-γ (79). Increased concentrations of IL-4 are believed to contribute to the development of equine asthma (80). Treatment for all species is limited to environmental management, anti-inflammatory therapy, and bronchodilator therapy. For horses, the use of corticosteroids, the mainstay of treatment, may be contraindicated in some patients. Inhaled corticosteroids, which reduce the risk of complications, are now available but may be cost prohibitive and inconvenient for some clients.

Equine asthma syndrome is similar to asthma seen in humans and cats, except equine asthma syndrome is dominated by neutrophil influx rather than eosinophil influx as it is in humans (77). Experimental studies using murine models of ovalbumin-induced asthma have shown that stem cells may be beneficial for managing the disease process. After one intravenous injection of human MSCs (1 × 10^6^ cells) in the mouse, there was a significant decrease in the presence of eosinophils in bronchial alveolar lavage fluid. A significant reduction in IL-5, IL-13, and IFN-γ, and a decrease in circulating IgE concentrations (81). Histologically, mice exhibited a decrease in airway inflammation, goblet cell hyperplasia, epithelial cell lining thickening, and collagen deposition. Similar results were shown in a ragweed-induced murine model of asthma (82). Intratracheal mouse MSC (5 × 10^4^ cells) administration has also shown to reduce bronchial hyperreactivity in a murine model (83).

Often the clinical patient has been showing signs of disease for longer periods of time, and the severity of changes at presentation may be higher than seen in experimental models. In a feline model of chronic allergic asthma, there was no difference in bronchial alveolar lavage cytology between stem cell-treated and control groups (84). Cats with experimentally-induced chronic asthma received intravenous allogeneic adipose derived MSCs (0.36–2.5 × 10^7^ MSCs/infusion) bimonthly for six treatments. Computed tomography (CT) was used to assess airway remodeling, and improvements were seen in the treated group at month eight, but this effect was not sustained. Repeated CT at month 12 showed no difference between the treated and control groups (84). These results suggest that multiple treatments with MSCs may be required to help manage chronic disease. The findings in an experimentally induced acute asthma trial were similar in that airway remodeling in MSC-treated cats (intravenous infusion of allogeneic MSCs) was significantly decreased compared to controls (85). There was also a decrease in airway eosinophilia and hyper-responsiveness in MSC treated cats compared to controls.

In humans, it is reported that MSCs act independent of the route of administration (intravenous v. intratracheal). Immunomodulatory effects are more predominant with intravenous injection compared to reparative mechanism being predominant with intratracheal administration (86). An *in vitro* study looking at the response of LPS-stimulated equine alveolar macrophages after exposure to conditioned medium and microvesicles from amniotic MSCs showed there was a significant decrease in TNF-α production in the groups treated with the MSC derivatives when compared to controls (87). These findings show that the amniotic MSC derivatives are capable of altering the alveolar macrophages cytokine release and may be able to play a role in treating inflammatory diseases affecting the equine lung.

To date, there are no published reports on the use of stem cells *in vivo* to treat equine asthma syndrome. While SCs may be beneficial in the treatment of asthma based on the murine models, the timing of their administration may play a role in response to treatment. The number of doses and follow-up treatment needs to be investigated.

## Gastrointestinal System and MSCs

Inflammatory bowel disease (IBD) is a broad term used to describe several small and large intestinal disorders in animals. In humans, IBD represents a specific disease process such as ulcerative colitis or Crohn's disease (88). The pathogenesis of IBD is thought to be inflammation mediated by the acquired immune system with an imbalance between the Th1 cells and proinflammatory cytokines overcoming the control mechanisms. An alternative theory proposes that it is a primary failure of regulatory lymphocytes and cytokines to control inflammation. New evidence suggests there is resistance to T cells undergoing apoptosis after activation (89), leading to perpetuation of the inflammation in Crohn's disease. The Th17 driven inflammatory response is also shown to play a role in the pathogenesis of IBD (90).

In small animals and horses, the clinical signs of IBD include diarrhea, weight loss, dependent edema, and lethargy which are associated with protein-losing enteropathy and malabsorption. The gastrointestinal (GI) submucosa and mucosa are infiltrated with eosinophils, plasma cells, lymphocytes, basophils, or macrophages (91). The etiology is often unknown and the type of cell that infiltrates the mucosa and submucosa affects prognosis. In dogs, Th17 cells are present in chronic inflammatory diseases and *in vitro* canine MSCs have an effect on the Th17 response (92). Comparing rectal biopsies between healthy control horses and IBD cases there was an increase in Th17-associated cytokines (93) suggesting that the Th17 response plays a role in the equine IBD disease process. In IBD, there is a breakdown of the normal mucosal barrier leading to increased gut permeability and increased exposure to commensal microbes (94). If MSCs are used for the treatment of this inflammatory disease, it is presumed that they will engraft in the mucosa of the gastrointestinal tract and be exposed to bacteria. In dogs it has been documented that there is significantly higher binding of IgG to gut bacteria in dogs with IBD compared to healthy dogs (95). IgG-coated bacteria from dogs with IBD had a greater macrophage TNF-α production. Interaction between stem cells and bacteria could alter the immune response. An *in vitro* canine model has shown that MSC behavior is altered by exposure to microbes, and the change in behavior is microbe dependent (96).

The current goals of treatment for humans with IBD are directed at relieving inflammation and treating signs and symptoms (97). Remission is hard to maintain, and patients often suffer from the side effects of drugs and surgeries (88). The only treatment available for horses at this time is corticosteroid therapy and the response is often poor (98). An effective immunomodulatory treatment without the complications of corticosteroids would be beneficial for horses.

IBD is influenced by interactions between genetics, environment, immune system, and microbial factors (99). In a murine model of colitis, treatment with human adipose-derived stem cells showed a dose-dependent improvement in survival and clinical signs. Down-regulation of the Th1 cytokine response also occurred with induction of regulatory T cell responses (100). In a DSS-induced colitis model in mice, three intravenous doses of induced MSCs or adipose derived MSCs (1 × 10^6^ cells/mouse) were administered 3 days apart and mice were still exposed to DSS during that time. MSC-treated mice showed significantly reduced clinical illness scores compared to untreated (101). Histologic appearance of the colon tissue showed a rapid recovery following MSC treatment and immunohistochemistry showed MSC treatment stimulated local angiogenesis and stimulated proliferation and recruitment of intestinal stem cells. After 10 days of MSC treatment, the composition of the microbiome became similar population to healthy, untreated mice (101). This suggests that there may be multiple independent effects of MSC treatment on the gastrointestinal tract. Stem cell therapy seems promising when examining studies using murine models of induced colitis, but these models do not account for the multifactorial nature of the disease and the severity of histopathologic changes that may be present in patients. Initial phase one trials of bone marrow derived stem cells for the treatment of refractory Crohn's disease in humans have produced mixed results. Some patients have shown improvement by reduction in their clinical assessment score, improvement in the mucosa on endoscopy, and reduction of inflammation and presence of cytokines on biopsy (88). However, the beneficial effects may be improved if the stem cells are given earlier in the course of the disease.

In feline patients with lymphocytic-plasmacytic enteritis, a single blinded randomized placebo-controlled study using allogeneic feline stem cells has been reported. No complications were seen from two intravenous injections (2 × 10^6^ cells/kg) of allogeneic MSCs 2 weeks apart and improvement in clinical signs was noted by owners (102). In a study of dogs diagnosed with moderate to severe IBD a single dose of allogeneic adipose derived stem cells (2 × 10^6^ cells/kg) was administered. No adverse reactions were observed, and clinical signs were improved up to 6 weeks after injection (103). Endoscopic and histologic improvements were still observed at a much later time point (108 days average) after a single infusion (104). Both of the small animal studies show potential for use when there is lymphocytic-plasmacytic infiltration. In horses, several types of IBD are distinguished based on histopathology. There can be granulomatous enteritis, multisystemic eosinophilic epitheliotropic disease, lymphocytic-plasmacytic enterocolitis, diffuse eosinophilic enterocolitis, and proliferative enteritis (91). The potential benefit of stem cells for other types of IBD is unknown.

Murine models have also shown other benefits of stem cells in the gastrointestinal system. Stem cells can accelerate gastric ulcer healing by honing to the site of injury. Labeled stem cells have been found only in the injured gastric mucosa and not in the normal mucosa (105). The route of administration for treatment of gastrointestinal disease is important. Mice that received intravenous stem cells showed a significant reduction in clinical and histopathologic severity when compared to mice that received intraperitoneal stem cells (106). Stem cells have also shown benefits when injected intravenously to attenuate peritoneal adhesions in experimentally induced lesions (107). The use of stem cells for treating gastric ulcers and preventing peritoneal or intestinal adhesions may become a further area of interest in the equine field.

## Equine Recurrent Uveitis and MSCs

Equine recurrent uveitis (ERU) has often been cited as the most common cause of blindness in horses. In the United States, prevalence has been reported between 2 and 25% (108). The true etiology and risk factors are not clear, but a genetic component as well as potential infectious etiologies (Leptospirosis) have been identified. Whatever the underlying etiology, it is well-established that ERU is an immune-mediated disorder characterized by recurrent episodes of inflammation (108). Immunohistochemistry has shown that the infiltrating cells in the ciliary body are lymphocytes and predominately T-cells with an increased transcription of IL-2 and IFN-γ (109). Th17 associated cytokines appear to play a role in equine recurrent uveitis (110).

Current treatment goals are to improve comfort and to reduce inflammation (111) with the aim of preserving vision. Topical administration of corticosteroid is the most common treatment, but long-term this treatment has side effects that include potentiation of infections, delayed epithelialization of corneal ulcers, and possible potentiation of calcific band keratopathy (108). Subconjunctival cyclosporine implants have been shown to reduce the duration and severity of inflammation, cellular infiltration, and decrease the production of pro-inflammatory cytokines (111). Not all horses are good candidates for this procedure and it also has potential complications.

With respect to MSC therapy, a study of recurrent autoimmune uveitis in rats examined long-term effects of different treatment regimens and compared the efficacy of MSCs to that of dexamethasone. Intravenous administration of bone marrow derived MSCs (5 × 10^6^ cells) for 3 consecutive days at the onset of the disease, reduced the inflammation during the peak of the attack and in the recovery phases. MSC treatments significantly reduced retinal damage and photoreceptor loss (112). MSC therapy may prove to be a better alternative to corticosteroid therapy in the management of this disease in horses.

Keratoconjunctivitis sicca is a common inflammatory condition of the eye in the dog. It has multiple potential etiologies, but it is thought to be immune mediated (113). One dose (1 × 10^6^ cells) of allogeneic adipose-derived stem cells was injected into the lacrimal glands of dogs affected with keratoconjunctivitis sicca. This produced resolution of clinical signs up to a year in mild to moderately affected eyes and severe eyes showed improvement in tear production (114). While the etiology is different from ERU, it is an immune-mediated condition of the eye. This small canine study suggests that MSCs could help modulate inflammation within the eye. The timing of administration does seem to be important as the less severely affected cases showed resolution of clinical signs.

There have been limited reports of MSCs being used to treat equine ocular diseases at this time. Equine immune mediated keratitis (IMMK) is a non-ulcerative corneal inflammatory disease characterized by a lymphocytic-plasmacytic infiltration of the cornea consistent with a dysregulated immune environment (115). Long-term topical therapy is often required and can be challenging with poor owner compliance and/or poor response to treatment. Subconjunctival administration of autologous bone marrow derived MSCs has been used in a small case series. Injection of 15 million MSCs was performed every 3–4 weeks for three to five injections (116). Three out of four horses showed positive response to therapy, including decreased opacity, irregularity, and vascularization of the cornea. Corneal disease remained stable for about 1 year after MSC treatment. These horses received additional topical treatments in conjunction with the MSCs, and there was no control group. The route of administration and timing of treatment may influence the response to treatment. Another IMMK case was reported to have a positive response to treatment after a single intravenous injection of blood-derived stem cells into the ophthalmic artery and topical stem cells administered three times daily for 2 weeks (117). While not an immune-mediated or inflammatory problem, equine stem cells have also been evaluated for corneal healing. Autologous bone marrow derived MSCs have shown *in vitro* to improve corneal wound healing (118).

## Safety and Limitations

A number of obstacles must be overcome before stem cells are more widely used. Stem cells are only beneficial for use in acute situations (such as endotoxemia, acute lung injury) if they can be administered immediately. The delay necessary for expansion of autologous stem cells may render their usefulness minimal. Another challenge is how *in vitro* results translate *in vivo*, as well as differences in response between the healthy horse and the clinically ill horse. Allogeneic stem cells may be more useful, because they can be administered almost immediately. Human and mice stem cells were originally believed to be immune privileged and poorly immunogenic, but murine studies have demonstrated that MHC class I and II mismatched MSCs elicit a robust immune response in allogeneic hosts with normal immune systems. Repeated challenge of MHC mismatched MSCs amplifies rejection (119). Murine MSCs are also capable of inducing a memory T-cell response after injection in immunocompetent hosts (120).

Safety of allogeneic MSC administration has been examined in horses. In a preliminary study of two horses, allogeneic equine MSCs were injected into lesions created in the superficial digital flexor tendon, and no signs of inflammatory reaction or immune rejection were seen (121). A single intra-articular injection of equine umbilical cord-derived stem cells (7.5 × 10^6^ cells) showed no significant difference in response between the joints injected with autologous or allogeneic stem cells (23). While a single injection appears to be safe, repeated intra-articular injections of allogeneic bone marrow derived MSCs caused an increase in total nucleated cell counts within the injected joint at the second injection. These changes did not occur in the autologous MSC treated joints after repeat injections (122). These findings suggest a local inflammatory response secondary to immune recognition after repeat intra-articular injections of allogeneic MSCs. Another study demonstrated that two intra-articular doses of allogeneic bone marrow derived MSCs in healthy joints did not have any complications or adverse reactions (123). Allogeneic MSCs from a single donor that was characterized as MHC-II negative demonstrated a low incidence of adverse reactions when injected into soft tissue structures (124).

Intradermal injections of allogeneic equine umbilical stem cells did not stimulate immediate or delayed hypersensitivity reactions following repeated intradermal injection (125). A different study showed that intradermal injection of allogeneic MSCs caused wheal formation in all horses and the horses also developed anti-ELA-A2 antibodies following injection of ELA-A2 MSCs (126). Intravenous injection of allogeneic MSCs (0.2 × 10^6^) showed no clinical adverse reactions in 291 horses. A small number of these horses received a second injection 6 weeks after the first with no reported complications (127). Three intravenous doses of allogeneic MSCs (25 × 10^6^) derived from adipose tissue or bone marrow administered 2 weeks apart showed no adverse effects in healthy horses (128). CD 8+ T cell numbers increased following repeated injection of bone marrow derived MSCs. Intravenous injection of equine cord blood derived MSCs in different carrier mediums did not elicit any changes in physical examination parameters, complete blood count, biochemistry profile, and coagulation profiles in ponies (129). Umbilical cord derived MSCs injected intramuscularly did not cause a significant inflammatory response. There was no difference in the muscle histopathology between MSC injections and controls (130).

Intravenous, intraarterial, and intralesional injection of equine allogeneic MSCs derived from both bone marrow and adipose tissue stimulated anti-MSC antibody development. The antibodies did diminish with time (42–600 days post-injections) in most horses (131). The highest level of antibody development was seen in horses with experimentally induced tendon lesion. This group of horses received the highest number of injections (four) and total number of MSCs (25–80 million cells/injection) (131). Despite the development of antibodies, no adverse clinical signs were reported. Development of cytotoxic ELA-A2 antibodies *in vivo* following incubation with allogeneic bone marrow derived MSCs has been reported (132). Evidence also exists that differentiation has an influence on equine MSC immunogenicity. In a study looking at tri-lineage differentiation of equine bone marrow derived-MSCs, osteogenesis and adipogenesis significantly upregulated MHC-I expression, but this did not occur in chondrogenesis. In all three lineages, MHC-II expression significantly increased (133). These finding suggest that differentiation may also play a role in the immune response and risk of allogeneic response. Allogeneic MSCs may elicit an immune response in the recipient, but how this may affect their therapeutic potential is unclear. Health status and other individual variables of the recipient may affect the strength of the individual immune response.

There is increasing evidence that variability exists among donors for MSCs and understanding the immune status of these donor cells prior to use will be essential. Equine MSCs are heterogenous in MHC-II expression, and there is even variability between passages and from different bone marrow isolates from the same horse (8). There may also be an ideal age range for the donor as it has been demonstrated that the number of MSCs within the system declines with age (11). MSCs isolated from older horses showed some degree of atypical morphology (8). Donor variation was also seen in the rate of proliferation, ability for cell passaging and trilineage differentiation in a small group of gender and age-matched horses (134).

Intravenous injection of allogeneic MSCs in cats with chronic kidney disease has produced complications that include vomiting and increased respiratory rate and effort (135). In other feline studies, five infusions of allogeneic MSCs from multiple donors was tolerated with no complications (85). Pre-activated allogeneic canine MSCs (2 × 10^6^ cells/kg body weight) have been administered intravenously every 2 weeks for three doses without complications (64). A dog being treated for hepatocutaneous syndrome received 46 infusions of 5 × 10^7^ allogeneic adipose derived stem cells over a 30 month period with no complications (136). These suggest that the safety of MSCs remains unclear and there may be a difference between the healthy and diseased patient. There also may be a difference between route of administration, MSC preservation and storage technique, and number of cells injected. A large number of human patients have currently received allogeneic mesenchymal stem cells for various diseases and have had no adverse events reported (137). Not all of those trials have evaluated development of antibodies. Importantly, human clinical studies in the United States now require the MSCs to be grown in media that contains no xenogeneic materials such as fetal bovine serum.

Traditionally, MSC expansion protocols use a medium supplemented with fetal bovine serum (FBS) to provide cells with nutrients, growth factors, and other beneficial proteins. MSCs incorporate foreign animal proteins during cell division and cell growth, and these xenogeneic proteins can elicit immune reactions. Evidence of antibodies against bovine proteins following MSC infusion is documented in humans (138, 139). Development of these antibodies may cause unfavorable immune responses at later injections. While the effect of development of antibodies against FBS has not been documented in horses, the use of xenogeneic cell growth media can alter the immunomodulatory response of MSCs. Equine and canine MSCs grown in a serum-free media secreted significantly less PGE_2_ than FBS-containing media (140). Intra-articular injection of autologous equine MSCs that were prepared in FBS demonstrated an inflammatory response when compared to autologous MSCs that had been through a FBS-depletion culture period prior to injection (122). This demonstrates that exposure to FBS in the horse may contribute to an immune reaction. However, it has been shown that some horses already had anti-bovine serum albumin (BSA) antibodies prior to MSC injection, and the titers did not change following MSC administration (131). The significance of inducing anti-FBS antibodies in horses remains unclear; however, xenogen-free cell growth media may be preferable to media containing FBS as the field goes forward. Furthermore, the use of FBS in previous immunologic studies muddies the waters with respect to allogeneic vs. autologous MSC use.

Determining the appropriate dose necessary for different applications is a challenge. A wide range of doses has been reported, from 10 × 10^6^ MSCs for tendon lesions up to 80 × 10^6^ cells systemically for immunomodulation (141). In humans, doses of 1–2 × 10^6^ cells/kilogram of body weight are being used in studies for immunomodulation in unhealthy patients (88) suggesting that the doses tested in horses may not be sufficient for the treatment of systemic inflammatory disease. The optimum timing of MSC injection remains unclear. If the local tissue environment into which they are injected influences MSCs, then the timing of the stem cell treatment during the acute or chronic phase of the disease may be important. In murine models of GI disease (101), respiratory disease (83), and sepsis (60), MSCs have been administered within hours of the inciting illness and have beneficial effects. A meta-analysis of large animal models of ischemic heart disease showed a trend toward better results when stem cell injection was performed over 1 week from the infarction (142). Spinal cord injuries in rats treated with intrathecal stem cells showed significant improvement when treated during the acute, subacute, and chronic phases of spinal cord injury (143). However, the greatest benefit was observed in the subacute group suggesting that the optimal timing is unclear and that is likely to vary among the conditions being treated. There are no current studies looking at timing of administration of MSCs for equine diseases. Finding a method to enhance the immunoregulatory properties of MSCs without affecting their immune evasive status could potentially improve allogeneic MSC therapeutic efficacy.

## Conclusions

Stem cell therapies may be efficacious in treating a wide range of conditions in the horse; however, a substantial research effort is needed. MSC therapeutic potential is highlighted by their unique properties: targeting of damaged tissues, inhibiting immune and inflammatory responses, and facilitating repair. This is only a small selection of the equine diseases that may benefit from the use of stem cells.

The high level of variation between studies with regard to: MSC tissue source, patient vs. donor source, cell isolation technique, cell culture technique, MSC activation status, MSC dose, route of administration, and dosing interval make comparing studies against each other difficult, if not entirely misleading. Further research is needed to test the safety and efficacy of these novel treatments in equines. This will require properly constructed clinical trials which are challenging to perform. Studies with appropriate MHC controls, and thorough analysis of the immune response are required. Additional challenges include identifying the optimal MSC donor phenotype and screening for all potential transmissible diseases. The dose of MSCs, route of administration, and number of doses required to treat or manage a disease will need to be established for each different condition, in addition to understanding the differences between autologous and allogeneic MSCs for treatment. The ideal timing of injection needs to be identified so that the environment may be able to promote the best activity of MSCs, but not so advanced that the tissues cannot be repaired or recovered.

MSCs have multiple pathways through which they achieve their immune suppression and anti-inflammatory roles, and the mechanisms are incompletely understood. Identifying the best way to activate the MSCs to get the most therapeutic potential will be essential. Trying to establish how MSCs will interact with other traditional treatments will also need to be evaluated.

Advances in stem cell biology and therapeutics have been substantial and continue to accelerate. The use of stem cells to enhance wound healing and to treat ischemic, cardiac, renal, and neurologic disease is being investigated and may find application in equine medicine. Proving safety, efficacy, and consistency will be essential to veterinarians and clients starting to accept the use of MSCs for treatment of systemic inflammatory diseases in horses. Most importantly, if stem cell therapy trials progress *without* optimizing all of the necessary parameters such as cell source, donor source, cell growth media, cell activation, route of administration, dose, timing and frequency of dosing, etc., the true potential of stem cell therapy may be thwarted due to perceived failure.

## Author Contributions

JB conceived of the review and created the outline. EM wrote the first draft of the manuscript. JB and EM edited the manuscript and approved the final version the manuscript.

### Conflict of Interest

The authors declare that the research was conducted in the absence of any commercial or financial relationships that could be construed as a potential conflict of interest.
